# Assessment of Ecological Quality Status in Shellfish Farms in South Korea Using Multiple Benthic Indices

**DOI:** 10.3390/ani15142086

**Published:** 2025-07-15

**Authors:** Se-Hyun Choi, Jian Liang, Chae-Woo Ma

**Affiliations:** 1 Fisheries Business Team, Korea Fisheries Infrastructure Public Agency, Seoul 08588, Republic of Korea; csh8723@fipa.or.kr; 2 Department of Biology, Soonchunhyang University, Asan 31538, Republic of Korea; 3 Department of Food Science and Engineering, Xinjiang Institute of Technology, Aksu 843000, China

**Keywords:** South sea of Korea, shellfish aquaculture, sustainable aquaculture, macrobenthos, *Anadara kagoshimensis*

## Abstract

South Korea is one of the world’s major centers for marine shellfish aquaculture. Since the industry’s rapid expansion began in the 1980s, concerns have grown regarding its environmental impacts on coastal marine ecosystems. Evaluating the benthic ecological quality status (EcoQs) of shellfish farms using benthic indices provides a scientific foundation for the sustainable management of aquaculture areas. In our study, five benthic indices (AZTI’s marine biotic index, BENTIX, benthic opportunistic polychaeta amphipoda index, benthic pollution index, and multivariate AMBI) and one composite index were selected to assess EcoQs of shellfish farms in Gangjin Bay, South Korea. Our results revealed significant differences in macrobenthic community structure and EcoQs between November and December in Gangjin Bay. Spearman correlation analysis and principal coordinates analysis (PCoA) demonstrated that the multivariate AMBI (M-AMBI) exhibited the best overall performance among indices. However, considering the ecological complexity, variability in farming practices, and site-specific conditions typical of shellfish aquaculture environments, the use of five benthic indices and a composite index is recommended to ensure a more comprehensive and robust evaluation of EcoQs in Korean shellfish farms.

## Simple Summary

Using benthic indices to assess shellfish farms’ ecological quality status (EcoQs) can provide valuable scientific support for their environmental monitoring, impact assessment, and sustainable management. In this study, we applied five benthic indices (AZTI’s marine biotic index, BENTIX, benthic opportunistic polychaeta amphipoda index, benthic pollution index, and multivariate AMBI) and one composite index to assess EcoQs of shellfish farms in Gangjin Bay, South Korea. Results revealed apparent monthly variations in EcoQs, with the multivariate AMBI (M-AMBI) showing the best overall performance among indices. However, considering the ecological complexity and spatial variability of shellfish farm environments, the use of all five benthic indices and the composite index is recommended to comprehensively and accurately assess EcoQs in aquaculture farms.

## 1. Introduction

With the continuous growth of the global population and worldwide decline of marine fishery resources, aquaculture has experienced significant expansion, intensification, and diversification in recent decades [1,2]. Aquaculture is now widely acknowledged as crucial for addressing the growing global demand for high-quality animal protein [3]. South Korea has become a major player in marine shellfish aquaculture as part of this global trend. In 2016, the country ranked fourth in global marine shellfish production, contributing approximately 15.7 million tons to the total global output [4]. Among the most significant aquaculture species in South Korea are *Crassostrea gigas*, *Ruditapes philippinarum*, and *Anadara kagoshimensis*, which dominate both in production volume and economic value [5]. Stimulated by sustained market demand and ongoing industry development, South Korea’s total aquaculture production has increased by nearly 300% over the past three decades. By 2018, the country’s aquaculture output had reached approximately 2.3 million tons, with shellfish accounting for around 18% of the total, equivalent to 417,000 tons [5].

Bivalve mollusks occupy a low trophic level in the food web. They perform feeding by filtering phytoplankton and detrital particles from seawater [6]. Although bivalve aquaculture is considered to have a lower environmental impact than finfish farming because it does not rely on external feed inputs, it can still have considerable effects on the benthic environment [7,8]. High-density bivalve aquaculture can produce large amounts of biodeposits, which may alter benthic community structure and degrade sediment conditions [9,10]. In addition, accumulating shells generated during farming can increase habitat complexity and heterogeneity [11].

Macrobenthos play a vital role in marine ecosystems’ energy flow and biogeochemical cycling. They are essential bioindicators for assessing marine ecosystem quality [12,13]. In shellfish farming, intensive cultivation practices and the accumulation of waste shells often result in elevated organic matter in sediments. This enrichment promotes the proliferation of opportunistic polychaetes, while leading to a significant decline in more sensitive taxa like amphipods [1,14]. Benthic indices based on macrobenthic community characteristics have been widely applied to assess the ecological quality status of aquaculture areas [15]. In Jiaozhou Bay, China, AMBI and M-AMBI have been proven to be effective in accurately evaluating the EcoQS of *Ruditapes philippinarum* farming grounds [1]. Although several studies in South Korea have recognized the potential of benthic indices for assessing ecological conditions in shellfish farming areas, most of them have relied on only one or two benthic indices [16,17]. Given the complexity of benthic ecosystems, relying on only one or two benthic indices might be insufficient to provide a comprehensive or accurate assessment of EcoQs [18].

Located in the South Sea of Korea, Gangjin Bay is one of the country’s major centers for shellfish production and export. However, the bay has undergone continuous environmental degradation since the large-scale expansion of shellfish aquaculture in the 1980s. Coupled with rising seawater temperatures, these changes have contributed to a steady decline in shellfish yields in recent years [19]. Although the benthic health index (BHI) has previously been used to assess the EcoQs of Gangjin Bay, it relies exclusively on polychaete community characteristics, overlooking other macrobenthic groups. As a result, BHI may not provide a comprehensive or accurate evaluation of EcoQs in benthic ecosystems with high species diversity [19].

Therefore, it is essential to accurately assess the EcoQs of Gangjin Bay to support future efforts to improve the environmental conditions of shellfish farming areas. Thus, the objective of this study was to conduct a comprehensive evaluation of EcoQs in shellfish aquaculture sites using a suite of benthic indices, including AZTI’s marine biotic index (AMBI) [20], BENTIX [21], benthic opportunistic polychaeta amphipoda index (BOPA) [22], benthic pollution index (BPI) [23], multivariate AZTI’s marine biotic index (M-AMBI) [24], and an integrated index. Findings of this study will provide a scientific basis for applying benthic indices in the monitoring and management of shellfish farming areas in South Korea.

## 2. Materials and Methods

### 2.1. Study Area

**Figure 1:**
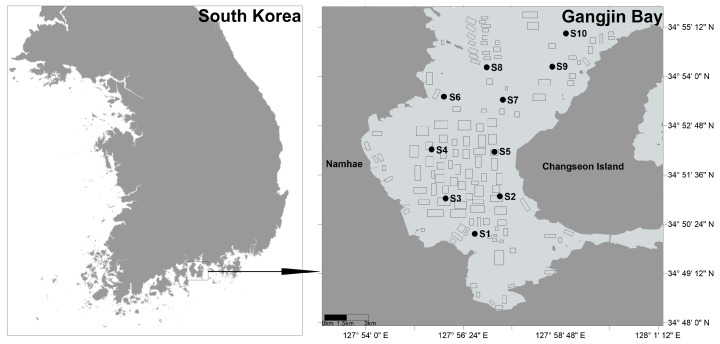
Sampling stations and shellfish aquaculture farms in Gangjin Bay of South Korea. Black rectangles on the right panel of the map indicate locations of shellfish farms.

Gangjin Bay, located in Namhae-gun, Jeollanam-do, South Korea, is one of the country’s major shellfish aquaculture sites (Figure 1). It extends approximately 10 km from east to west and 18 km from north to south. The bay has an average depth of 8.9 m, a maximum depth of 24 m, and a total area of approximately 200 km^2^. Within the bay, 182 shellfish aquaculture farms occupy a combined area of 11.18 km^2^, accounting for about 5.6% of the bay’s total surface area. The remaining area consists largely of tidal flats and subtidal zones that serve as important habitats for macrobenthic organisms and support ecosystem services such as nutrient cycling and nursery grounds for fish. In 2016, the total shellfish production was approximately 3300 tons in Gangjin Bay [19]. The primary bivalve species cultured in Gangjin Bay is *Anadara kagoshimensis*, while *Crassostrea gigas* is also farmed, particularly in the northern part of the bay. It is seeded in April each year and harvested annually in October and November using bottom trawl nets.

### 2.2. Sample Collection

Ten sampling stations in Gangjin Bay were surveyed during spring tide periods of November and December 2022. All ten sampling stations are located in or near shellfish farming areas (Figure 1). Coordinates of the ten sampling stations are presented in Table S1.

At each station, macrobenthic samples were collected using 0.05 m^2^ Van Veen grab samplers. Two replicate grabs were taken to ensure sampling reliability. Collected sediments were sieved onboard through a 1 mm mesh to separate macrozoobenthic organisms. Retained specimens were initially fixed in 10% buffered formalin to preserve morphological features and later transferred to 80% ethanol for long-term storage and analysis.

In addition, sediment samples were collected in duplicate at each station using the same grab samplers and stored in a deep freezer for subsequent analysis. Bottom water samples were collected using a Niskin water sampler. Water temperature, salinity, dissolved oxygen (DO), and pH were measured onboard using a YSI Pro2030 multiparameter instrument (YSI Inc., Yellow Springs, OH, USA). Remaining seawater samples were stored in a deep freezer for further laboratory analysis.

### 2.3. Sample Analysis

#### 2.3.1. Seawater Sample Analysis

Suspended solids were measured using the gravimetric method with glass fiber filters (GF/F). Chlorophyll-a was extracted using 90% acetone and analyzed according to the Turner fluorometric method as specified in the Marine Environmental Process Test Guidelines issued by the National Institute of Fisheries Science (2010) [25].

#### 2.3.2. Sediment Sample Analysis

Ignition loss (IL) was determined by placing approximately 20 g of wet sediment into a pre-weighed crucible, drying it at 110 °C for over 24 h until a constant weight was reached and then combusting it at 550 °C for 2 h in a furnace. For particle size analysis, organic matter in each sample was removed using 30% hydrogen peroxide. The pretreated sediment was then separated into coarse and fine fractions using a 4 phi (ϕ) (0.063 mm) sieve. A portion of the fine fraction was analyzed with a laser particle size analyzer (Mastersizer 2000, Malvern Instruments Ltd., Malvern, UK), while the coarse fraction was further classified and weighed using a series of sieves ranging from 0 phi (ϕ) to 4 phi (ϕ) in 1 phi (ϕ) intervals. Chemical oxygen demands (CODs) in both seawater and sediment were measured via titration using potassium dichromate as an oxidizing agent, following guidelines of the National Institute of Fisheries Science (2010) [25]. Acid volatile sulfide (AVS) concentrations in sediments were assessed using the same standards as a gas detection tube method. Total organic carbon (TOC) was quantified by treating approximately 0.2 g of dried sediment with 5 mL of 0.1 N HCl to remove inorganic carbon. Samples were then sequentially dried at 70 °C for 6 h and at 105 °C for 2 h before analysis using an elemental analyzer. For analysis of heavy metals (As, Cd, Cr, Cu, Ni, Pb, and Zn), dried sediment samples were finely ground using a mortar and pestle. About 0.1 g of each sample was digested with concentrated nitric, perchloric, and hydrofluoric acids in a PTFE vessel on a hot plate at 150 °C for 24 h. After drying, the residue was redissolved in 1–2% nitric acid. Heavy metal concentrations were determined using inductively coupled plasma optical emission spectrometry (ICP-OES, Avio 200, PerkinElmer Inc., Waltham, MA, USA).

#### 2.3.3. Macrobenthic Sample Analysis

In the laboratory, macrobenthic samples were examined under a Leica M80 stereomicroscope (Leica Microsystems GmbH, Wetzlar, Germany) and identified to the lowest possible taxonomic level, preferably to species. All individuals were counted. Identified specimens were preserved in 80% ethanol solution.

### 2.4. Data Analysis

#### 2.4.1. Benthic Indices

Since the study area is located within a bay, we selected five benthic indices (AMBI, BENTIX, BOPA, BPI, and M-AMBI) widely used in previous studies to assess EcoQs of Korean bays [26] (Table 1). The AMBI classifies macrobenthic species into five ecological groups based on their tolerance to organic pollution [20]. The BENTIX operates on a similar principle to AMBI but simplifies the classification into three ecological groups [21]. The BOPA is based on the ratio of opportunistic polychaetes to amphipods within the macrobenthic community [22]. The BPI is the only one among the four indices that is specifically developed for the Yellow Sea ecosystem. It classifies benthic organisms based on their feeding types [26]. The M-AMBI is based on the AMBI and Shannon’s diversity index, with species richness integrated through factor analysis [24]. AMBI and M-AMBI indices were calculated using the AMBI 6.0 software, with the species database updated to the October 2024 version (https://ambi.azti.es/, accessed on 1 January 2025). The classification of opportunistic polychaetes in the BOPA index was based on the AMBI species database. Functional groupings used in the BPI index were derived from our previous studies [27,28]. Species not listed in the AMBI database were classified according to our previous studies and by referring to functional groupings defined in the BPI [27,28]. When fewer than four macrobenthic species are identified at a sampling station, the EcoQs is classified as “bad”.

#### 2.4.2. Composite Indices

To facilitate marine environmental management, the EcoQs of the five benthic indices were simplified. Stations classified as high or good were considered to have acceptable benthic ecological quality, while those rated as moderate, bad, or poor were classified as having unacceptable benthic ecological quality [28] (Table S2).

Composite indices were derived from EcoQs of the five benthic indices, aiming to resolve inconsistencies that might have arisen when multiple indices are used simultaneously [23,29]. The calculation method is presented in Table 2.

#### 2.4.3. Statistical Analysis

To evaluate characteristics of environmental factors in Gangjin Bay, principal component analysis (PCA) was conducted. Effects of environmental variables on benthic community structure were assessed using distance-based linear modeling (DistLM). Spearman’s rank correlation analysis was employed to examine relationships among ecological indices or between these indices and environmental variables. To control for the false discovery rate in multiple testing, the Benjamini–Hochberg FDR correction was applied to the *p*-values obtained from Spearman’s correlation analyses. Principal coordinates analysis (PCoA) based on Bray–Curtis dissimilarity was used to evaluate differences in community composition among EcoQs as classified by benthic indices. Cohen’s Kappa coefficient analysis was applied to assess the consistency of EcoQs’ classifications among different indices. Agreement levels based on kappa values are presented in Table S3. To evaluate the monthly differences in the values of the benthic indices and the composite index, normality was first assessed using the Shapiro–Wilk test, followed by the application of the Mann–Whitney U analysis. Differences in macrobenthic community structure between stations classified as having acceptable and unacceptable EcoQs were assessed using PERMANOVA analysis.

Spearman’s correlation with false discovery rate (FDR) correction, Kappa analyses, Shapiro–Wilk test, and Mann–Whitney U analysis were performed using R software (version 4.3.0) (https://cran.r-project.org/bin/windows/base/ accessed on 1 January 2025). PCA, DistLM, PERMANOVA, and PCoA were conducted in PRIMER version 7 with the PERMANOVA+ add-on (PRIMER-e Ltd., Auckland, New Zealand).

## 3. Results

### 3.1. Environmental Characteristics

Environmental data for Gangjin Bay are presented in Table 3. Average concentrations of heavy metals in sediments (in ascending order) were as follows: Cd (0.294 mg/kg), Cu (7.203 mg/kg), As (8.575 mg/kg), Ni (24.315 mg/kg), Zn (41.125 mg/kg), Cr (64.775 mg/kg), and Pb (77.36 mg/kg). AVS and SS exhibited high coefficients of variation, with values of 0.71 and 0.63, respectively.

**Figure 2:**
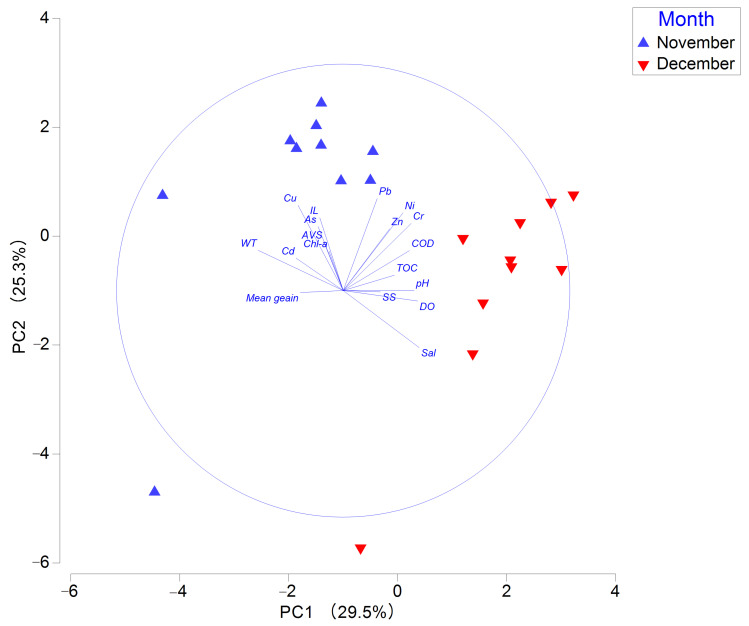
Principal component analysis plot of environmental factors in Gangjin Bay. Note: AVS, acid-volatile sulfide; COD, chemical oxygen demand; Chl-a, chlorophyll-a; TOC, total organic carbon; IL, ignition loss.

In the principal component analysis, the first two axes (PC1 and PC2) together explained 54.8% of the total variance in environmental variables. Water temperature, salinity, and DO were major eigenvectors associated with PC1, while Cu and Pb were dominant eigenvectors on PC2 (Table S4). Sampling stations in November were positioned in the upper part of the PC2 axis and the left side of the PC1 axis compared to those in December. This indicates distinct differences between the two months regarding Cu and Pb concentrations, water temperature, and salinity (Figure 2).

### 3.2. Macrobenthic Compositions

**Figure 3:**
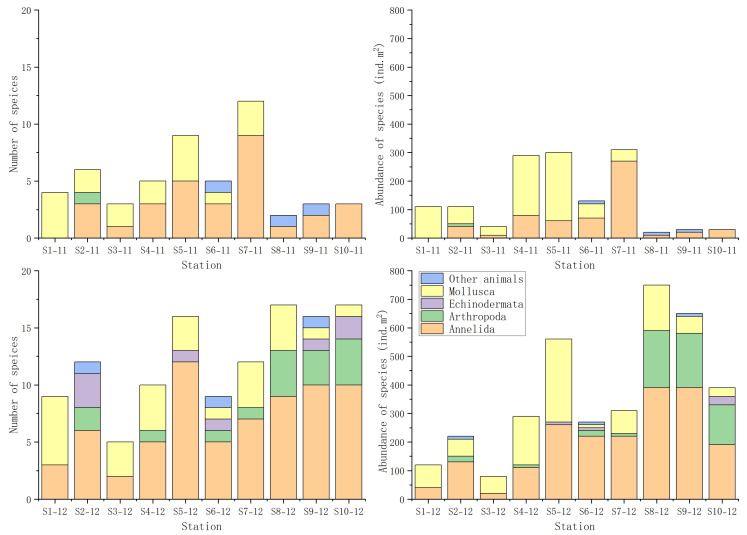
Species compositions and abundance at each station. Note: ind, individual.

A total of 49 macrobenthic species were identified during the two sampling campaigns conducted in November and December 2022. These included 28 Annelida, 10 Mollusca, 7 Arthropoda, 1 Nemertea, and 1 Sipuncula. The lowest species richness and abundance were recorded at Station 8 in November. Only two species were identified at this Station in November, showing a density of just 20 ind.m^2^. In contrast, the highest species richness was observed at Station 8 and Station 10 in December, each with 17 species. The highest abundance was also recorded at Station 8 in December, reaching 750 ind.m^2^ (Figure 3) (Table S5).

### 3.3. Results of Benthic and Composite Indices

**Figure 4:**
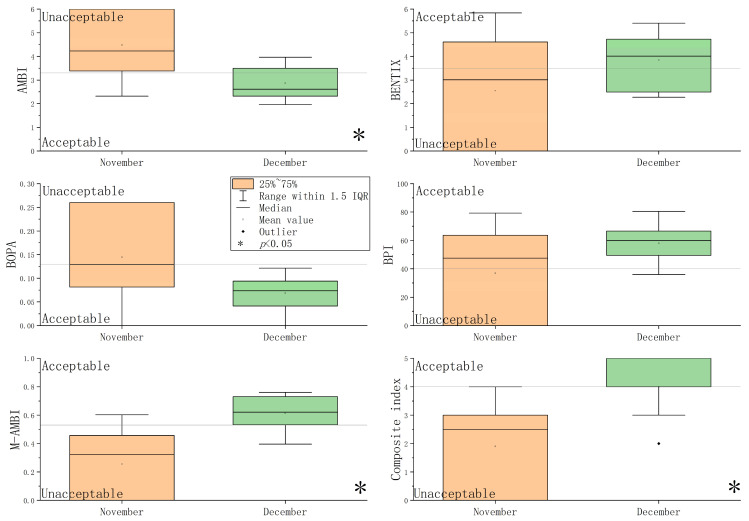
Box plot of the five benthic indices and their corresponding EcoQs.

Values of the five benthic indices and their corresponding EcoQs at each station are shown in Figure S1 and Figure 4. In Stations 3, 8, and 10 during November, only three species were recorded. EcoQs were evaluated as “bad” by five benthic indices. Based on the five benthic indices, EcoQs in November were poorer than those in December. The Mann–Whitney U analysis revealed that the values of AMBI, M-AMBI, and the composite index differed significantly between November and December (*p* < 0.05) (Figure 4).

**Figure 5:**
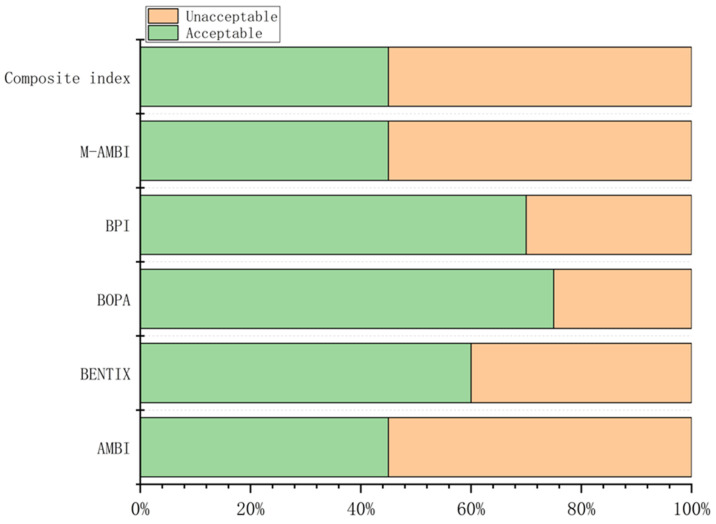
Percentages of acceptable and unacceptable EcoQS determined by the five benthic indices and the composite index.

Values of the composite index and corresponding ecological quality status (EcoQs) at each station are shown in Table S6. According to BOPA, 75% of the stations were assessed as having an acceptable EcoQ, while only 45% were classified as acceptable by AMBI, M-AMBI, and the composite index (Figure 5).

### 3.4. Results of Statistical Analysis

Results of the DistLM analysis showed that dissolved oxygen and suspended solids had significant effects on the macrobenthic community (Table 4).

Spearman correlation analysis showed that AMBI, BOPA, M-AMBI, and the composite index were significantly correlated. In addition, M-AMBI was correlated with the composite index (all *p* < 0.05) (Table S7).

AMBI was significantly correlated with water temperature, DO, and pH. BOPA was significantly correlated with DO. M-AMBI exhibited significant correlations with water temperature, salinity, DO, and pH. The composite index was significantly correlated with water temperature, salinity, DO, and pH (all *p* < 0.05) (Table S7).

Kappa analysis revealed that the agreement between AMBI or M-AMBI and the composite index in assessing EcoQs was very good. In contrast, AMBI and BENTIX showed null agreement with EcoQs assessments. Overall, the level of agreement among most benthic indices, except for AMBI, M-AMBI, and the composite index, was generally low (Table 5).

**Figure 6:**
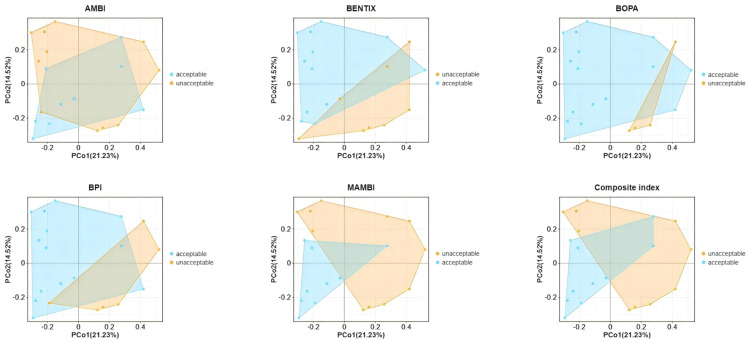
PcoA analysis based on macrobenthic abundance.

In the PCoA plot (Figure 6), BENTIX and M-AMBI demonstrated a clear capacity to distinguish between samples of acceptable and unacceptable ecological quality, indicating their effectiveness in capturing differences in macrobenthic community structure within shellfish farming areas of Gangjin Bay. In contrast, indices such as AMBI, BOPA, BPI, and the composite index showed noticeable overlaps between acceptable and unacceptable regions, suggesting a more limited ability to reflect variations in benthic community compositions under specific environmental conditions of Gangjin Bay.

PERMANOVA analysis revealed that the macrobenthic community structures significantly differed between stations classified as having acceptable and unacceptable EcoQs based on BOPA (F = 1.99, *p* = 0.02), BPI (F = 2.06, *p* = 0.02), M-AMBI (F = 2.81, *p* = 0), and the composite index (F = 2.03, *p* = 0.01).

## 4. Discussion

### 4.1. Macrobenthic Community Compositions in Gangjin Bay

In our study, species richness and abundance of macrobenthos in November were lower than those in December (Figure 3). In Gangjin Bay, shellfish farms used bottom trawl nets to collect shellfish in October and November. Bottom trawling has a negative impact on species density and biodiversity of benthic communities, particularly affecting larger macrobenthic organisms with body lengths greater than 4 mm [30]. DistLM analysis indicated that DO was the most influential environmental factor affecting macrobenthic communities in the study area. In the bays of the South Sea of Korea, hypoxia in bottom waters is a common phenomenon primarily caused by eutrophication and limited water circulation due to a semi-enclosed geomorphology [31]. Furthermore, PCA analysis revealed a distinct difference in DO level between November and December, which might explain the observed temporal variation in macrobenthic community structure.

### 4.2. Performances of Benthic and Composite Indices

Although five indices and the composite index indicated that EcoQs of Gangjin Bay in December were better than those in November, the kappa analysis revealed substantial discrepancies among different indices at each sampling station. Among the six indices, BENTIX showed low or very low agreements with all other indices (Table 5). At Stations 1, 2, and 9 in December, BENTIX was the only index that assessed the EcoQs as unacceptable. BENTIX was initially designed for oligotrophic marine environments of the Eastern Mediterranean, where it could categorize both tolerant and opportunistic species within the same ecological group (Group II) [21]. While this approach might be appropriate for ecosystems with relatively low anthropogenic pressure, its applicability in more impacted environments becomes limited [31,32]. In particular, when tolerant species dominate the benthic assemblage, the index’s ability to detect anthropogenic impacts is diminished, potentially leading to an underestimation of EcoQs [33,34].

Spearman correlation analysis revealed that the benthic indices and the composite index exhibited limited responsiveness to organic matter and heavy metals (Table S6). Although these indices are traditionally developed based on the tolerance of macrobenthic organisms to organic enrichment and their feeding strategies [23,35], organic pollution is generally associated with predictable ecological responses, including changes in trophic structure, feeding behavior, and species composition driven by physiological tolerance [36,37]. However, in this study, such responses may have been obscured due to the timing of sampling, which occurred after shellfish harvesting activities involving benthic trawling. In contrast, the ecological effects of heavy metals are usually sublethal and primarily associated with genotoxicity, which may not immediately lead to observable changes in benthic community structure [38,39,40]. Compared to other benthic indices, M-AMBI responded to the greatest number of environmental variables. This can be attributed to the fact that M-AMBI incorporates both macrobenthic diversity and species richness in its calculation, which enhances its ability to reflect complex environmental gradients and ecological changes [41].

Overall, M-AMBI responded to the most significant environmental variables and achieved the most distinct separation between acceptable and unacceptable stations in the PCoA analysis and PERMANOVA analysis of macrobenthic communities. Other studies have also supported the superior performance of M-AMBI to other benthic indices [42,43,44]. In contrast, the BENTIX index appeared to be less suitable for assessing ecological EcoQs of shellfish farms in Gangjin Bay.

### 4.3. Use Benthic Indices in Shellfish Farms

In South Korea, the BHI is commonly used to assess the EcoQs of aquaculture areas. BHI was developed based on the Infaunal Trophic Index (ITI). It incorporates ecological grouping of benthic polychaetes based on their response to organic matter content in sediments, categorizing them into four ecological groups [17]. Although BHI requires identification of only polychaeta species, its focus on a single taxonomic group, similar to the BOPA index used in this study, restricts its applicability to specific environmental contexts. For example, the BOPA index was initially developed to assess the effects of oil spill pollution. It might not adequately capture broader ecological variations in other disturbance scenarios [45,46]. Relying on a single index to assess EcoQs of aquaculture areas, particularly shellfish farming sites, appears unreliable. Each benthic index is developed based on distinct theoretical foundations, classification criteria, or specific types of environmental disturbances [42,47]. As a result, these indices might have limitations in fully capturing the complexity and heterogeneity of benthic ecosystems in aquaculture environments. As demonstrated in this study, indices that depend heavily on a single taxonomic group might yield unreliable results when that group is underrepresented. This limitation was especially evident in regions such as Gangjin Bay, where polychaetes accounted for less than 50% of the macrobenthic community at several stations.

In this study, the M-AMBI index demonstrated comparatively better performance in assessing EcoQs. However, as discussed above, no single benthic index is unlimited, particularly in complex, multi-stressor environments like aquaculture zones. Therefore, we recommend using multiple benthic indices (AMBI, BENTIX, BOPA, BPI, and M-AMBI) in combination with a composite index to obtain a more robust and accurate assessment of ecological conditions at shellfish farming sites in South Korea. It is also important to note that ecological groupings used in the AMBI index were initially developed based on European coastal ecosystems [20]. When applying AMBI or M-AMBI to Korean coastal environments, recalibration of these groupings, particularly the categorization of opportunistic species, is necessary to improve the accuracy and regional relevance of the assessment [48].

### 4.4. Recommendations for Shellfish Farm Management

In Gangjin Bay, Pb concentrations at several major monitoring stations have already exceeded the Threshold Effect Level (TEL), with Cd concentrations at some locations approaching this threshold (Table S8) [49]. Moreover, average concentrations of As and Pb in Gangjin Bay are higher than those observed in the East Sea of Korea and Dandong Bay [26,27] (Table S8). Correlation analysis revealed that Cu sedimentary concentrations were negatively correlated with bottom water salinity, suggesting that Cu was primarily derived from terrestrial inputs (Table S7). Since bivalves can directly absorb heavy metals from the surrounding aquatic environment [50,51], they are susceptible to metal contamination in areas with elevated sedimentary metal levels. Furthermore, bioaccumulation and biomagnification of trace metals within the aquatic food web can increase the potential risk of exposure to higher trophic organisms, including humans [52,53,54]. Considering that Pb concentrations are above the TEL in Gangjin Bay sediments, regular monitoring of heavy metal concentrations in farmed shellfish from this region is necessary to assess potential environmental risks and ensure food safety.

The hydrodynamic regime is a key environmental factor affecting the distribution and retention of particulate and dissolved wastes from aquaculture operations, shaping deposition patterns of biodeposits generated by bivalve cultivation [55,56]. Gangjin Bay exhibits relatively low water current velocities as a semi-enclosed bay, limiting the natural dispersal of aquaculture-derived wastes. Therefore, it is necessary to control the stocking density of shellfish within the bay. To mitigate environmental impacts, implementing integrated multi-trophic aquaculture (IMTA) systems is preferred over monoculture practices [57]. Additionally, relocating shellfish farms toward outer areas of the bay might help enhance waste dispersion and reduce localized accumulation [58]. Due to funding and human resources limitations, this study only conducted two surveys during the winter season. However, these periods coincided with post-harvest conditions and intensified bottom trawling activities, which are known to significantly disturb benthic habitats. As such, the results may also reflect the impact of anthropogenic disturbances on EcoQs. There were notable differences in EcoQs between the two surveys in the same sampling area. Therefore, a comprehensive year-round investigation of Gangjin Bay is necessary to better understand seasonal dynamics of EcoQs in shellfish farming areas. Such data are essential for developing scientifically sound and effective management strategies for sustainable aquaculture in the region.

## 5. Conclusions

Based on two survey cruises in Gangjin Bay, this study revealed notable monthly variations in macrobenthic species richness and abundance. EcoQs in November were worse than those in December. While Spearman correlation analysis and PCoA supported the superior performance of the M-AMBI to other benthic indices, Kappa analysis highlighted inconsistencies in EcoQs assessments among different indices at individual sampling stations. Given the ecological complexity of shellfish farming environments in Korea, along with the variability in aquaculture practices and site-specific conditions, we recommend using a combination of five benthic indices (AMBI, BENTIX, BOPA, BPI, and M-AMBI) and the composite index to achieve a more comprehensive and robust evaluation of ecological quality in shellfish farms.

Furthermore, in light of the observed environmental conditions and known ecological challenges associated with shellfish aquaculture in Gangjin Bay, potential management strategies such as reducing farming density, adopting integrated multi-trophic aquaculture (IMTA), and considering the relocation of farms to more open coastal areas may warrant further investigation.

## Figures and Tables

**Table 1:** Computational methods and threshold values used to assess EcoQs based on benthic indices (adapted from Liang et al., 2024 [23]).

Indices	Algorithm	Index Values	EcoQs	Reference
AMBI	$\begin{array}{l} =[(0\times \%\;EGI)+(1.5\times \%\;EGII)+(3\times \%\;EGIII)\\ +(4.5\times \%\;EGIV)(6\times \%\;EGV)]/100 \end{array}$	0.0–1.2 1.2–3.3 3.3–5.0 5.0–6.0 >6.0	High Good Moderate Poor Bad	[20]
BENTIX	=[6×% G1 +2(%G2+% G3)]/100	6–4.5 4.5–3.5 3.5–2.5 2.5–2.0 0.0	High Good Moderate Poor Bad	[21]
BOPA	=log[(fP)/(fA+1)+1)]	0–0.02452 0.02455–0.13002 0.13002–0.19884 0.19884–0.25512 >0.25512	High Good Moderate Poor Bad	[22]
BPI	$\begin{array}{l} =[1-(a\times N1+b\times N2+c\times N3+d\times N4)/(N1+\\ N2+N3+N4)/d]\times 100 \end{array}$	60–100 40–60 30–40 20–30 0–20	High Good Moderate Poor Bad	[23]
M-AMBI	$=K+(a\times AMBI)+\left(b\times H^{\prime }\right)+(c\times S)$	>0.77 0.53–0.77 0.38–0.53 0.20–0.38 ≤0.2	High Good Moderate Poor Bad	[24]

Notes: For AMBI, EGI = disturbance-sensitive species; EGII = disturbance-indifferent species; EGIII = disturbance-tolerant species; EGIV = second-order opportunistic species; EGV = first-order opportunistic species. For BENTIX, GI = EGI + EGII; GII = EGIII + EGIV; GIII = EGV. For BOPA, fP = the frequency of opportunistic polychaetes; fA = the frequency of amphipods. For BPI, N1 = filter feeders or large carnivores; N2 = surface deposit feeders or small carnivores; N3 = subterranean deposit feeders; N4 = opportunistic species; a = 0; b = 1; c = 2; d = 3. For M-AMBI, S = number of species; H′ = Shannon’s diversity index; a, b, and c = coefficients.

**Table 2:** Methodology for calculating the composite index and threshold criteria used to determine the final ecological quality (adapted from Liang et al., 2024 [23]).

Composite Index	Assessment	Final Ecological Quality
0	All indices have assessed the ecological quality of the station as unacceptable.	Unacceptable
1	Four indices have assessed the ecological quality of the station as unacceptable.	Unacceptable
2	Three indices have assessed the ecological quality of the station as unacceptable.	Unacceptable
3	Two indices have assessed the ecological quality of the station as unacceptable.	Unacceptable
4	One index has assessed the ecological quality of the station as unacceptable.	Acceptable
5	Five indices have assessed the ecological quality of the station as acceptable.	Acceptable.

**Table 3:** Range of environmental data in subtidal zones of Gangjin Bay.

Environment Data	Range (min–max) (November)	Range (min–max) (December)	Mean ± CV (November)	Mean ± CV (December)	Mean ± CV (Total)
AVS, mg/g	0.003–0.25	0.03–0.22	0.13 ± 0.71	0.09 ± 0.66	0.11 ± 0.71
COD, mg/g	16.79–32.55	16.28–31.93	21.99 ± 0.19	25.84 ± 0.17	23.92 ± 0.19
DO, mg/L	6.13–9.16	7.90–8.67	7.22 ± 0.13	8.23 ± 0.03	7.72 ± 0.11
TOC, mg/g	8.42–14.46	6.18–14.41	12.14 ± 0.14	12.11 ± 0.20	12.12 ± 0.17
IL, %	8.00–9.60	7.60–8.90	8.88 ± 0.06	8.26 ± 0.06	8.57 ± 0.07
Mean grain size, ∮	7.70–8.70	7.50–8.60	8.20 ± 0.05	7.94 ± 0.05	8.07 ± 0.05
pH	7.94–8.15	8.10–8.32	8.05 ± 0.01	8.14 ± 0.01	8.10 ± 0.01
Salinity, PSU	29.90–30.30	30.90–30.90	30.03 ± 0.004	30.90 ± 0	30.47 ± 0.01
Suspended solids, mg/L	3.08–25.44	5.40–29.48	9.48 ± 0.66	10.70 ± 0.64	10.09 ± 0.63
Water temperature, °C	16.80–18.40	6.30–9.10	17.42 ± 0.03	7.92 ± 0.11	12.67 ± 0.39
Chl-a, μg/L	1.24–3.40	0.95–2.86	2.25 ± 0.35	1.85 ± 0.29	2.05 ± 0.34
As, mg/kg	7.40–12.60	5.30–9.20	9.55 ± 0.19	7.60 ± 0.16	8.58 ± 0.21
Cd, mg/kg	0.09–0.71	0.02–0.39	0.39 ± 0.58	0.20 ± 0.71	0.29 ± 0.71
Cr, mg/kg	40.90–65.20	40.90–84.20	60.07 ± 0.12	69.48 ± 0.19	64.78 ± 0.17
Cu, mg/kg	5.45–10.10	5.19–6.77	8.22 ± 0.15	6.18 ± 0.08	7.20 ± 0.19
Ni, mg/kg	14.10–25.20	14.80–31.60	23.03 ± 0.14	25.60 ± 0.19	24.32 ± 0.18
Pb, mg/kg	22.00–99.60	25.60–90.80	79.54 ± 0.27	75.18 ± 0.25	77.36 ± 0.26
Zn, mg/kg	35.30–43.30	37.20–45.10	40.08 ± 0.06	41.45 ± 0.07	41.13 ± 0.07

Note: AVS, acid-volatile sulfide; COD, chemical oxygen demand; Chl-a, chlorophyll-a; TOC, total organic carbon; IL, ignition loss.

**Table 4:** DistLM results revealing effects of environmental data on macrobenthic community structure.

Environmental Data	Pseudo-F	*p*	Proportion of Variation Explained
AVS, mg/g	1.074	0.095	0.080
COD, mg/g	1.121	0.320	0.059
DO, mg/L	2.409	0.002	0.118
TOC, mg/g	0.900	0.498	0.048
IL, %	1.277	0.222	0.066
Mean grain size, ∮	0.798	0.692	0.042
pH	1.270	0.245	0.066
Salinity, PSU	1.682	0.060	0.085
Suspended solids, mg/L	1.816	0.037	0.092
Water temperature, °C	1.633	0.067	0.083
Chl-a, μg/L	0.605	0.869	0.033
As, mg/kg	1.561	0.095	0.080
Cd, mg/kg	1.593	0.061	0.081
Cr, mg/kg	0.864	0.623	0.046
Cu, mg/kg	1.598	0.069	0.082
Ni, mg/kg	0.942	0.506	0.050
Pb, mg/kg	1.332	0.171	0.069
Zn, mg/kg	0.770	0.744	0.041

Note: AVS, acid-volatile sulfide; COD, chemical oxygen demand; Chl-a, chlorophyll-a; TOC, total organic carbon; IL, ignition loss.

**Table 5:** Results of kappa analysis.

Indices	Kappa Value	Level of Agreement
AMBI/BENTIX	−0.078	Null
AMBI/BOPA	0.429	Moderate
AMBI/BPI	0.327	Low
AMBI/M-AMBI	0.596	Good
AMBI/Composite index	0.798	Very Good
BENTIX/BOPA	0.222	Low
BENTIX/BPI	0.348	Low
BENTIX/M-AMBI	0.118	Very low
BENTIX/Composite index	0.118	Very low
BOPA/BPI	0.375	Low
BOPA/M-AMBI	0.238	Low
BOPA/Composite index	0.429	Moderate
BPI/M-AMBI	0.327	Low
BPI/Composite index	0.327	Low
M-AMBI/Composite index	0.798	Very Good

## Data Availability

The raw data supporting the conclusions of this article will be made available by the authors on request.

## Supplementary Materials

The following supporting information can be downloaded at https://www.mdpi.com/article/10.3390/ani15142086/s1, Table S1: Coordinates of sampling stations; Table S2: Standards for categorising benthic biotic index values as acceptable or unacceptable in assessing ecological quality; Table S3: The threshold for kappa value.; Table S4: Eigenvectors of environmental factors with PC1 and PC2; Table S5: Species list in Gangjin Bay; Table S6: Values of composite index at each station; Table S7: Result of Spearman’s correlation analysis. Table S8: Comparison of heavy metal concentrations (mg/kg) and average concentrations in sediments between the Gangjin Bay and other areas [59]; Figure S1: Value of the five benthic indices and their corresponding EcoQs at each station.

## References

[1] Wang L., Fan Y., Yan C., Gao C., Xu Z., Liu X. (2017). Assessing Benthic Ecological Impacts of Bottom Aquaculture Using Macrofaunal Assemblages. Mar. Pollut. Bull..

[2] Zhang J., Hansen P.K., Wu W., Liu Y., Sun K., Zhao Y., Li Y. (2020). Sediment-Focused Environmental Impact of Long-Term Large- Scale Marine Bivalve and Seaweed Farming in Sungo Bay, China. Aquaculture.

[3] Cressey D. (2009). Aquaculture: Future Fish. Nature.

[4] Peng D., Hou X., Li Y., Mu Y. (2019). The Difference in Development Level of Marine Shellfish Industry in 10 Major Producing Countries. Mar. Policy.

[5] Park J., Shin S.K., Wu H., Yarish C., Yoo H.I., Kim J.K. (2021). Evaluation of Nutrient Bioextraction by Seaweed and Shellfish Aquaculture in Korea. J. World Aquac. Soc..

[6] Lercari D., Bergamino L. (2011). Impacts of Two Invasive Mollusks, Rapana Venosa (Gastropoda) and Corbicula Fluminea (Bivalvia), on the Food Web Structure of the Río de La Plata Estuary and Nearshore Oceanic Ecosystem. Biol. Invasions.

[7] Walls A.M., Kennedy R., Edwards M.D., Johnson M.P. (2017). Impact of Kelp Cultivation on the Ecological Status of Benthic Habitats and Zostera Marina Seagrass Biomass. Mar. Pollut. Bull..

[8] Sun X., Filgueira R., Wang N., Guyondet T., Dong J., Zhang X. (2023). Assessing Shellfish Farming-Mediated Benthic Impacts Based on Organic Carbon Flux Simulation and Composition of Macrofaunal Community. Sci. Total Environ..

[9] Callier M.D., McKindsey C.W., Desrosiers G. (2008). Evaluation of Indicators Used to Detect Mussel Farm Influence on the Benthos: Two Case Studies in the Magdalen Islands, Eastern Canada. Aquaculture.

[10] Miron G., Landry T., Archambault P., Frenette B. (2005). Effects of Mussel Culture Husbandry Practices on Various Benthic Characteristics. Aquaculture.

[11] Sanchez-Jerez P., Krüger L., Casado-Coy N., Valle C., Sanz-Lazaro C. (2019). Mollusk Shell Debris Accumulation in the Seabed Derived from Coastal Fish Farming. J. Mar. Sci. Eng..

[12] Rehitha T.V., Vineetha G., Madhu N.V. (2022). Ecological Habitat Quality Assessment of a Tropical Estuary Using Macrobenthic Functional Characteristics and Biotic Indices. Environ. Sci. Pollut. Res..

[13] Islam S.S., Samanta S., Mahato S., Bhattacharya S., Midya S. (2024). Diversity of Meiobenthic Fauna in Costal Environment. Spatial Modeling of Environmental Pollution and Ecological Risk.

[14] Liao Y., Liu Q., Shou L., Tang Y., Liu Q., Zeng J., Chen Q., Yan X. (2022). The Impact of Suspended Oyster Farming on Macrobenthic Community in a Eutrophic, Semi-Enclosed Bay: Implications for Recovery Potential. Aquaculture.

[15] Borja Á., Rodríguez J.G., Black K., Bodoy A., Emblow C., Fernandes T.F., Forte J., Karakassis I., Muxika I., Nickell T.D. (2009). Assessing the Suitability of a Range of Benthic Indices in the Evaluation of Environmental Impact of Fin and Shellfish Aquaculture Located in Sites across Europe. Aquaculture.

[16] Jung R.-H., Seo I.-S., Choi M., Park S.R., Choi B.-M., Kim M.H., Kim Y.J., Yun J.S. (2014). Community Structure and Health Assessment of Macrobenthic Assemblages during Spring and Summer in the Shellfish Farming Ground of Wonmun Bay, on the Southern Coast of Korea. Kor. J. Fish. Aquat. Sci..

[17] Jung R.H., Yoon S.-P., Park S., Hong S.-J., Kim Y.J., Kim S. (2023). Introduction to the Benthic Health Index Used in Fisheries Environment Assessment. J. Korean Soc. Mar. Environ. Saf..

[18] Dong J.-Y., Sun X., Zhang Y., Zhan Q., Zhang X. (2021). Assessing Benthic Habitat Ecological Quality Using Four Benthic Indices in the Coastal Waters of Sanshandao, Laizhou Bay, China. Ecol. Indic..

[19] Kim S., Yoon S.-P., Park S., Jung R.H. (2024). Patterns in Benthic Polychaete Community and Benthic Health Assessment at Longline and Bottom Culture Shellfish Farms in Gangjin Bay, Namhae, Korea. J. Korean Soc. Mar. Environ. Saf..

[20] Borja A., Franco J., Pérez V. (2000). A Marine Biotic Index to Establish the Ecological Quality of Soft-Bottom Benthos Within European Estuarine and Coastal Environments. Mar. Pollut. Bull..

[21] Simboura N., Zenetos A. (2002). Benthic Indicators to Use in Ecological Quality Classification of Mediterranean Soft Bottom Marine Ecosystems, Including a New Biotic Index. Medit. Mar. Sci..

[22] Dauvin J.C., Andrade H., de-la-Ossa-Carretero J.A., Del-Pilar-Ruso Y., Riera R. (2016). Polychaete/Amphipod Ratios: An Approach to Validating Simple Benthic Indicators. Ecol. Indic..

[23] Liang J., Ma C.-W., Kim K.-B., Son D.-S. (2024). Can the Ecological Quality of Several Bays in South Korea Be Accurately Assessed Using Multiple Benthic Biotic Indices?. J. Mar. Sci. Eng..

[24] Muxika I., Borja Á., Bald J. (2007). Using Historical Data, Expert Judgement and Multivariate Analysis in Assessing Reference Conditions and Benthic Ecological Status, According to the European Water Framework Directive. Mar. Pollut. Bull..

[25] National Institute of Fisheries Science (2010). National Institute of Fisheries Science Notification of Marine Environmental Process Test Standards 2010.

[26] Liang J., Ma C.-W., Kim K.-B. (2024). Comparing the Environmental Impacts of Pollution from Two Types of Industrial Zones on the Coast. Front. Mar. Sci..

[27] Liang J., Huang H.-R., Ma C.-W., Son D.-S., Kim S.-K. (2024). Using the Heavy Metal Indices and Benthic Indices to Assess the Ecological Quality in the Tidal Flats of Garolim Bay, South Korea. Water.

[28] Blanchet H., Lavesque N., Ruellet T., Dauvin J.C., Sauriau P.G., Desroy N., Desclaux C., Leconte M., Bachelet G., Janson A.-L. (2008). Use of Biotic Indices in Semi-Enclosed Coastal Ecosystems and Transitional Waters Habitats—Implications for the Implementation of the European Water Framework Directive. Ecol. Indic..

[29] Maghsoudlou A., Momtazi F., Hashtroudi M.S. (2020). Ecological Quality Status (EcoQs) of Chabahar Sub-Tropical Bay Based on Multimetric Macrobenthos-Indexes Approach: Response of Bio-Indexes to Sediment Structural/Pollutant Variables. Reg. Stud. Mar. Sci..

[30] McLaverty C., Eigaard O.R., Gislason H., Bastardie F., Brooks M.E., Jonsson P., Lehmann A., Dinesen G.E. (2020). Using Large Benthic Macrofauna to Refine and Improve Ecological Indicators of Bottom Trawling Disturbance. Ecol. Indic..

[31] Lee J., Park K.-T., Lim J.-H., Yoon J.-E., Kim I.-N. (2018). Hypoxia in Korean Coastal Waters: A Case Study of the Natural Jinhae Bay and Artificial Shihwa Bay. Front. Mar. Sci..

[32] Onyena A.P., Nkwoji J.A., Chukwu L.O. (2023). Sediment Characteristics and Ecological Quality Evaluation of a Brackish Creek Using AZTI’s Marine Biotic and Bentix Indices. Aquat. Sci..

[33] Leshno Y., Benjamini C., Edelman-Furstenberg Y. (2016). Ecological Quality Assessment in the Eastern Mediterranean Combining Live and Dead Molluscan Assemblages. Mar. Pollut. Bull..

[34] Dias H.Q., Sukumaran S., Srinivas T., Mulik J. (2018). Ecological Quality Status Evaluation of a Monsoonal Tropical Estuary Using Benthic Indices: Comparison via a Seasonal Approach. Environ. Sci. Pollut. Res..

[35] Liang J., Huang H.-R., Shu M.-Y., Ma C.-W. (2025). Assessing the Impact of Land-Based Anthropogenic Activities on the Macrobenthic Community in the Intertidal Zones of Anmyeon Island, South Korea. Land.

[36] Souza F.M.D., Gilbert E.R., Camargo M.G.D., Pieper W.W. (2013). The Spatial Distribution of the Subtidal Benthic Macrofauna and Its Relationship with Environmental Factors Using Geostatistical Tools: A Case Study in Trapandé Bay, Southern Brazil. Zoologia.

[37] Bon M., Grall J., Gusmao J.B., Fajardo M., Harrod C., Pacheco A.S. (2021). Functional Changes in Benthic Macrofaunal Communities along a Natural Gradient of Hypoxia in an Upwelling System. Mar. Pollut. Bull..

[38] Souza F.M., Gilbert E.R., Brauko K.M., Lorenzi L., Machado E., Camargo M.G. (2021). Macrobenthic Community Responses to Multiple Environmental Stressors in a Subtropical Estuary. PeerJ.

[39] Nunes S.M., Josende M.E., Ruas C.P., Gelesky M.A., Júnior F.M.R.D.S., Fattorini D., Regoli F., Monserrat J.M., Ventura-Lima J. (2017). Biochemical Responses Induced by Co-Exposition to Arsenic and Titanium Dioxide Nanoparticles in the Estuarine Polychaete Laeonereis Acuta. Toxicology.

[40] Liang J., Ma C.-W., Kim K.-B. (2024). Ecological Risk Assessment of Heavy Metals in Surface Sediments and Their Impact on Macrobenthos in Asan Bay, South Korea. Front. Mar. Sci..

[41] Dong J.-Y., Wang X., Zhang X., Bidegain G., Zhao L. (2023). Integrating Multiple Indices Based on Heavy Metals and Macrobenthos to Evaluate the Benthic Ecological Quality Status of Laoshan Bay, Shandong Peninsula, China. Ecol. Indic..

[42] Pinto R., Patrício J., Baeta A., Fath B.D., Neto J.M., Marques J.C. (2009). Review and Evaluation of Estuarine Biotic Indices to Assess Benthic Condition. Ecol. Indic..

[43] Tian S., Liao Y., Tang Y., Liu Q., Zhang R., Shou L., Zeng J. (2023). Assessment of Macrobenthic Communities of Rocky Intertidal Zone from Zhejiang Offshore Islands with AZTI Marine Biotic Index. Ecol. Indic..

[44] Liang J., Ma C.-W., Kim K.-B. (2025). Assessment of Benthic Ecological Quality Status in the Subtidal Zone of Northern Jeju Island, South Korea, During Summer Based on Macrobenthos. Animals.

[45] Sukumaran S., Mulik J., Rokade M.A., Kamble A. (2014). Impact of ‘Chitra’ Oil Spill on Tidal Pool Macrobenthic Communities of a Tropical Rocky Shore (Mumbai, India). Estuaries Coasts.

[46] Mulik J., Sukumaran S., Jisna M.J., Rao M.N. (2023). Tracing the Impact and Recovery Trajectory of Oil Spill Affected Tropical Rocky Intertidal Macrobenthic Communities Using the BOPA Index. Mar. Pollut. Bull..

[47] Salas F., Marcos C., Neto J.M., Patrício J., Pérez-Ruzafa A., Marques J.C. (2006). User-Friendly Guide for Using Benthic Ecological Indicators in Coastal and Marine Quality Assessment. Ocean Coast. Manag..

[48] Gillett D.J., Weisberg S.B., Grayson T., Hamilton A., Hansen V., Leppo E.W., Pelletier M.C., Borja A., Cadien D., Dauer D. (2015). Effect of Ecological Group Classification Schemes on Performance of the AMBI Benthic Index in US Coastal Waters. Ecol. Indic..

[49] Ministry of Oceans and Fisheries (2018). Korea Marine Seawater Quality Standard. https://www.mof.go.kr/en/index.do.

[50] Jeong H., Byeon E., Kim D.-H., Maszczyk P., Lee J.-S. (2023). Heavy Metals and Metalloid in Aquatic Invertebrates: A Review of Single/Mixed Forms, Combination with Other Pollutants, and Environmental Factors. Mar. Pollut. Bull..

[51] Pavón A., Riquelme D., Jaña V., Iribarren C., Manzano C., Lopez-Joven C., Reyes-Cerpa S., Navarrete P., Pavez L., García K. (2022). The High Risk of Bivalve Farming in Coastal Areas with Heavy Metal Pollution and Antibiotic-Resistant Bacteria: A Chilean Perspective. Front. Cell. Infect. Microbiol..

[52] Saidon N.B., Szabó R., Budai P., Lehel J. (2024). Trophic Transfer and Biomagnification Potential of Environmental Contaminants (Heavy Metals) in Aquatic Ecosystems. Environ. Pollut..

[53] Ali H., Khan E. (2019). Trophic Transfer, Bioaccumulation, and Biomagnification of Non-Essential Hazardous Heavy Metals and Metalloids in Food Chains/Webs—Concepts and Implications for Wildlife and Human Health. Human Ecol. Risk Assess. An Int. J..

[54] Qiu Y.-W. (2015). Bioaccumulation of Heavy Metals Both in Wild and Mariculture Food Chains in Daya Bay, South China. Estuar. Coast. Shelf Sci..

[55] Silva E., Garbossa L.H.P., Nuñer A.P.O., Lapa K.R. (2019). Hydrodynamic Modelling of the Dispersion and Deposition of Biodeposits from Marine Bivalve Mollusc Farming under Neap and Spring Tides in Santa Catarina Island Bays. Aquaculture.

[56] Zhou Y., Zhang S., Liu Y., Yang H. (2014). Biologically Induced Deposition of Fine Suspended Particles by Filter-Feeding Bivalves in Land-Based Industrial Marine Aquaculture Wastewater. PLoS ONE.

[57] Strand Ø., Jansen H.M., Jiang Z., Robinson S.M.C. (2019). Perspectives on Bivalves Providing Regulating Services in Integrated Multi-Trophic Aquaculture. Goods and Services of Marine Bivalves.

[58] Holmer M. (2010). Environmental Issues of Fish Farming in Offshore Waters: Perspectives, Concerns and Research Needs. Aquacult. Environ. Interact..

[59] Liang J., Ma C.-W., Son D.-S. (2024). Using the Heavy Metal and Biotic Indices to Assess Ecological Quality in the Central Area of the East Sea, South Korea. Water.

