# Supplementary material for: Assessment of Ecological Quality Status in Shellfish Farms in South Korea Using Multiple Benthic Indices
Source: Animals (Basel). 2025 Jul 15;15(14):2086. doi: 10.3390/ani15142086 (PMC12292003; doi:10.3390/ani15142086)
Supplement: Supplementary file 1 [file animals-15-02086-s001.zip › Supplementary Material.pdf]

Supplementary Material

Assessment of Ecological Quality Status in Shellfish Farms in South Korea Using Multiple Benthic Indices

Table S1. Coordinates of sampling stations.

Sampling site	Latitude	Longitude
S1	34°50'16.50" N	127°56'33.39" E
S2	34°51'3.67"N	127°57'24.04 E
S3	34°51'21.80"N	127°55'54.22" E
S4	34°52'36.90"N	127°55'44.12" E
S5	34°52'25.17"N	127°57'6.75"E
S6	34°53'44.05"N	127°56'19.19" E
S7	34°53'23.68" N	127°57'50.50" E
S8	34°54'25.65" N	127°57'22.86"E
S9	34°54'14.82"N	127°58'49.50"E
S10	34°55'5.32"N	127°59'19.82"E

Table S2. Standards for categorising benthic biotic index values as acceptable or unacceptable in assessing ecological quality.

**Indices**	**Thresholds**	**Acceptable, Unacceptable**
AMBI	≤3.3	Acceptable
	>3.3	Unacceptable
BENTIX	≥3.5	Acceptable
	<3.5	Unacceptable
BOPA	≤0.13002	Acceptable
	>0.13002	Unacceptable
BPI	≥40	Acceptable
	<40	Unacceptable
M-AMBI	≥0.53	Acceptable
	<0.53	Unacceptable

Table S3. The threshold for kappa value.

Kappa values	The level of agreement
≤ 0.05	Null
0.051–0.20	Very low
0.21–0.40	Low
0.41–0.55	Moderate
0.56–0.70	Good
0.71–0.85	Very good
0.86–0.99	Almost perfect
1.0	Perfect

Table S4. Eigenvectors of environmental factors with PC1 and PC2.

Environment Factors	PC1	PC2
AVS, mg/g	-0.085	0.213
COD, mg/g	0.293	0.177
DO, mg/L	0.329	-0.046
TOC, mg/g	0.226	0.068
IL, %	-0.109	0.321
Mean grain size, ∮	-0.189	-0.009
pH	0.313	0.001
Salinity, PSU	0.335	-0.252
Suspended Solids, mg/L	0.164	-0.006
Water temperature, ℃	-0.375	0.178
Chl-a, μg/L	-0.063	0.175
As, mg/kg	-0.111	0.283
Cd, mg/kg	-0.208	0.143
Cr, mg/kg	0.3	0.297
Cu, mg/kg	-0.199	0.377
Ni, mg/kg	0.264	0.343
Pb, mg/kg	0.15	0.407
Zn, mg/kg	0.205	0.273

Note: AVS, acid-volatile sulfide; COD, chemical oxygen demand; TOC, total organic carbon; IL, ignition loss.

Table S6. Values of composite index at each station.

Station	Composite index	EcoQs
S1-11	3	Unacceptable
S2-11	4	Acceptable
S3 -11	1	Unacceptable
S4 -11	3	Unacceptable
S5 -11	3	Unacceptable
S6 -11	2	Unacceptable
S7 -11	3	Unacceptable
S8-11	0	Unacceptable
S9-11	0	Unacceptable
S10-11	0	Unacceptable
S1-12	4	Acceptable
S2-12	4	Acceptable
S3-12	2	Unacceptable
S4-12	3	Unacceptable
S5-12	4	Acceptable
S6-12	4	Acceptable
S7-12	5	Acceptable
S8-12	5	Acceptable
S9-12	4	Acceptable
S10-12	5	Acceptable

Table S7. .Result of Spearman's correlation analysis.

**Variable 1**	**Variable 2**	**Spearman R**	***P*-value**	**FDR Corrected *P***
AMBI	BOPA	0.6375	0.0025	0.0332
AMBI	M-AMBI	-0.7905	0	0.0028
AMBI	Composite index	-0.7681	0.0001	0.0041
AMBI	Wt	0.6861	0.0008	0.0163
AMBI	DO	-0.6634	0.0014	0.0226
AMBI	pH	-0.6541	0.0018	0.0261
BOPA	DO	-0.6316	0.0028	0.0356
M-AMBI	Composite index	0.9020	0	0
M-AMBI	Wt	-0.7199	0.0003	0.0097
M-AMBI	Sal	0.6817	0.0009	0.0168
M-AMBI	DO	0.7409	0.0002	0.0067
M-AMBI	pH	0.6443	0.0022	0.0304
Composite index	Wt	-0.7034	0.0005	0.0114
Composite index	DO	0.7099	0.0005	0.0113
Composite index	pH	0.6268	0.0031	0.0373
Wt	Sal	-0.7071	0.0005	0.0113
Wt	DO	-0.7667	0.0001	0.0041
Wt	pH	-0.7602	0.0001	0.0042
Sal	IL	-0.6707	0.0012	0.0204

Sal	Cu	-0.7300	0.0003	0.0082
Cr	Ni	0.9864	0	0
Cr	Zn	0.6159	0.0038	0.0441

Table S8. Comparison of heavy metal concentrations (mg/kg) and average concentrations in sediments between the Gangjin Bay and other areas

Study area	As	Cd	Cr	Cu	Pb	Zn	Ni	Reference
Gangjin Bay	5.30-12.60 (8.85)	0.02-0.71 (0.29)	40.9-84.20 (64.78)	5.19-10.10 (7.20)	22.00-99.60 （77.36）	35.30-45.10 （41.13）	14.10-31.60 （24.32）	This study
East Sea of Korea	1.91-6.14 (3.35)	0.04-0.33 (0.124)	8.71-66.98 (43.7)	1.83-32.56 (16.6)	9.79-32.27 (20.48)	11.27-109.31 (65.9)	NA	Liang et al., 2024a
Dangdong Bay, Korea	0.30-0.70 (0.48)	1.40-3.30 (2.56)	NA	13.90-22.50 (17.17)	35.80-60.00 (50.06)	67.90-84.90 (76.10)	NA	Liang et al., 2024b
Threshold Effect Level	14.50	0.75	116	20.6	44.00	64.40	47.2	Ministry of Oceans and Fisheries 2018
Probable Effect Level	75.5	2.72	181	64.4	119	157	80.5	Ministry of Oceans and Fisheries 2018

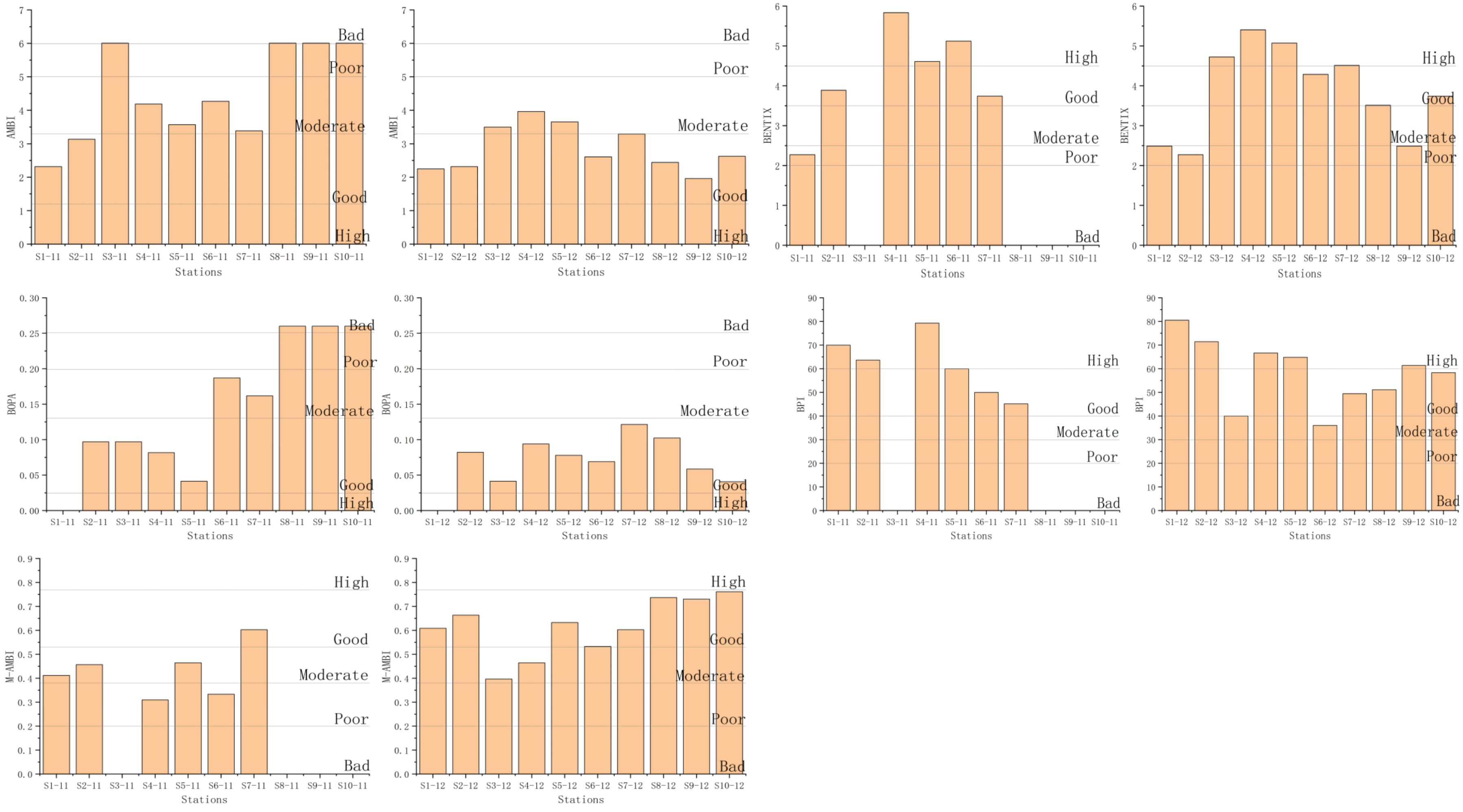

Figure S1. Value of the five benthic indices and their corresponding EcoQs at each station.

Reference

Liang, J., Ma, C.-W. & Son, D.-S. Using the Heavy Metal and Biotic Indices to Assess Ecological Quality in the Central Area of the East Sea, South Korea. Water 16, 1230 (2024a).

Liang, J., Ma, C.-W. & Kim, K.-B. Comparing the environmental impacts of pollution from two types of industrial zones on the coast. Front. Mar. Sci. 11, 1433536 (2024b).

Ministry of Oceans and Fisheries, Korean Marine Seawater Quality Standard. (2018).
